# Investigating the Link between Intermediate Metabolism, Sexual Dimorphism, and Cardiac Autonomic Dysfunction in Patients with Type 1 Diabetes

**DOI:** 10.3390/metabo14080436

**Published:** 2024-08-06

**Authors:** María Rosa Insenser, Lía Nattero-Chávez, Manuel Luque-Ramírez, Sara de Lope Quiñones, Alejandra Quintero-Tobar, Sara Samino, Núria Amigó, Beatriz Dorado Avendaño, Tom Fiers, Héctor F. Escobar-Morreale

**Affiliations:** 1 Diabetes, Obesity and Human Reproduction Research Group, Instituto Ramón y Cajal de Investigación Sanitaria (IRYCIS) & Centro de Investigación Biomédica en Red Diabetes y Enfermedades Metabólicas Asociadas (CIBERDEM), Universidad de Alcalá, 28034 Madrid, Spain; mariarosa.insenser@salud.madrid.org (M.R.I.); manuel.luque@salud.madrid.org (M.L.-R.); sara.lope@salud.madrid.org (S.d.L.Q.); mariaalejandra.quintero@salud.madrid.org (A.Q.-T.); hectorfrancisco.escobar@salud.madrid.org (H.F.E.-M.); 2 Department of Endocrinology and Nutrition, Hospital Universitario Ramón y Cajal, 28034 Madrid, Spain; bdorado@salud.madrid.org; 3 Biosfer Teslab, CIBERDEM, 43206 Tarragona, Spain; saminosara@gmail.com (S.S.); nuriaamigo@gmail.com (N.A.); 4 Institut d’Investigació Sanitària Pere Virgili (IISPV), Department of Basic Medical Sciences, Universitat Rovira i Virgili (URV), 43002 Tarragona, Spain; 5 Laboratory for Hormonology and Department of Endocrinology, Ghent University Hospital, 9000 Ghent, Belgium; tom.fiers@uzgent.be

**Keywords:** autonomic nervous system, cardioautonomic neuropathy, cardiac autonomic neuropathy, low molecular weight metabolites, metabolomics, sexual dimorphism, type 1 diabetes mellitus

## Abstract

Sexual dimorphism influences cardiovascular outcomes in type 1 diabetes (T1D), with women facing a higher relative risk of macrovascular events compared to men, especially after menopause. This study hypothesizes that abnormalities in intermediate metabolism may be associated with cardiac autonomic neuropathy (CAN) in T1D. We aim to assess low molecular weight metabolites (LMWM) as markers of CAN in T1D, considering the effects of sexual dimorphism and age. In this cross-sectional study, we included 323 subjects with T1D (147 women and 176 men), with a mean age of 41 ± 13 years. A total of 44 women and 41 men were over 50 years old. CAN was assessed using Ewing’s tests, and serum metabolites were analyzed by proton nuclear magnetic resonance spectroscopy (^1^H-NMR). Patients with CAN had lower levels of valine, isoleucine, and threonine, and higher levels of lactate, compared to those without CAN. These differences persisted after adjusting for BMI and estimated glucose disposal rate (eGDR). In a logistic regression model (R² = 0.178, *p* < 0.001), the main determinants of CAN included isoleucine [Exp(β) = 0.972 (95% CI 0.952; 0.003)], age [Exp(β) = 1.031 (95% CI 1.010; 1.053)], A_1c_ [Exp(β) = 1.361 (95% CI 1.058; 1.752)], and microangiopathy [Exp(β) = 2.560 (95% CI 1.372; 4.778)]. Sex influenced LMWM profiles, with over half of the metabolites differing between men and women. However, no interactions were found between CAN and sex, or between sex, age, and CAN, on metabolomics profiles. Our findings suggest an association between CAN and LMWM levels in T1D. The sexual dimorphism observed in amino acid metabolites was unaffected by the presence of CAN.

## 1. Introduction

Sexual dimorphism influences cardiovascular outcomes in patients with type 1 diabetes (T1D), with women facing a higher relative risk of macrovascular events compared to men, particularly after menopause [1]. Cardiac autonomic dysfunction (CAN) is prevalent among individuals with T1D. Such a disruption of the delicate balance of the autonomic nervous system, which regulates cardiovascular function, escalates the risk of adverse cardiac events and mortality [2,3]. Although the impact of sex on CAN remains unclear, our previous findings suggested a sex difference in the prevalence of CAN among patients with T1D, which might contribute to the abovementioned sex disparity in cardiovascular disease [4,5]. This highlights the need to thoroughly explore the underlying mechanisms and factors contributing to CAN in T1D.

The autonomic nervous system links the brain to the peripheral organs and is responsible for nutrient partitioning [6]. Circulating intermediate metabolism—including low molecular weight metabolites (LMWM) such as fatty acids and amino acids—are indicative of the metabolic activity across all cells and tissues in the body. Amino acids, beyond their role as building blocks of proteins and polypeptides, are crucial in regulating key metabolic pathways, gene expression, protein phosphorylation, and hormone synthesis. Fatty acids serve as both a metabolic energy source and important signaling molecules. LMWM plays a crucial role in autonomic regulation [7,8,9] aside from its influence on glucose metabolism. Dysregulation of fatty acids and amino acids may disrupt autonomic balance, altering heart rate (HR) variability [8,9]. Over the past two decades, proton nuclear magnetic resonance (^1^H-NMR) has emerged as a reliable and effective method for the simultaneous evaluation of numerous circulating metabolites with efficiency and robustness. This technology is particularly useful for assessing metabolic activity and inflammation in different diseases with underlying metabolic mechanisms, making it highly valuable for clinical research and health monitoring. Recent advancements, such as the identification of LMWM as early predictors of metabolic dysregulation, offer new avenues for exploration of the role of intermediate metabolism in the development of CAN. Therefore, this study aims to investigate, in adult patients with T1D, the potential role of energy homeostasis-related metabolites and amino acids as markers of CAN. Additionally, we will explore the influence of sexual dimorphism and age on LMWM levels in patients with T1D and autonomic dysfunction. Our objective adopts a diagnostic perspective, focusing on identifying markers that could simplify the diagnosis of CAN or identify patients with T1D who are at risk and should be screened for CAN. This understanding could guide personalized clinical management strategies for these patients.

## 2. Materials and Methods

### 2.1. Study Population

In a cross-sectional study, we recruited 345 consecutive adult patients with T1D who attended regularly our diabetes outpatient clinic at an Academic Hospital in Madrid, Spain (ClinicalTrials.gov Identifier: NCT04950634). Recruitment occurred between January 2018 and December 2021. Eligible patients met the following criteria for T1D diagnosis: (a) history of ketoacidosis and/or diabetic autoimmunity, and (b) dependence on insulin for survival according to the American Diabetes Association guidelines [10]. Exclusion criteria included: (a) age ≥ 85 years; (b) inability to complete or comprehend CAN assessment; (c) diabetic foot; (d) end-stage renal disease or undergoing renal replacement therapy; (e) current pregnancy; and (f) diagnosis of diabetes mellitus types other than T1D. Ultimately, 323 patients agreed to participate and provided signed informed consent.

### 2.2. Assessment of Anthropometric, Biochemical, and Clinical Variables

We conducted a thorough review of medical history and current medications, focusing on clinical parameters associated with T1D at the time of recruitment. Subsequently, all study participants underwent a comprehensive anthropometric evaluation.

Microvascular complications, including T1D-related eye disease, neuropathy (defined as any T1D-related neurological complication), and nephropathy (defined as any T1D-related kidney disease), were recorded. Additionally, macrovascular complications such as cerebrovascular disease, coronary artery disease, and peripheral arterial disease were assessed. We also evaluated all patients for diabetic peripheral neuropathy [11] through detailed clinical history and clinical tests using a 128 Hz tuning fork for vibration perception, ankle reflexes, and a 10 g monofilament test.

Estimated glucose disposal, (eGDR), a validated measure of insulin sensitivity, was calculated as follows: eGDR (mg/kg/min) = 24.395 − (12.971 × waist-to-hip ratio) − (3.388 × hypertension) − (0.601 × A_1c_) [12]. Samples for sex steroid measurement were immediately centrifuged, and aliquots of serum and plasma were separated, coded, and frozen at −80 °C until thawed for analysis. We analyzed total testosterone (T), sex hormone-binding globulin (SHBG), luteinizing hormone (LH), follicle-stimulating hormone (FSH), and estradiol (E_2_) in serum samples. Serum total T and E_2_ were measured by LC–MS/MS at the Laboratory of Clinical Biology of the University of Ghent, Belgium, using an AB Sciex 6500 triple-quadrupole mass spectrometer (AB Sciex, Toronto, Canada). The lower limit of quantification (LLOQ) was 1.2 ng/dL (0.04 nmol/L) for total T and the interassay CV was 8.3% at 36.7 ng/dl (1.27 nmol/L) and 3.1% at 307.8 ng/dl (10.68 nmol/L). Serum LLOQ was <0.5 pg/mL (1.9 pmol/L) for E2 and the interassay CV was 4.0% at 21 pg/mL (77 pmol/L). SHBG was assayed by an automated immunochemiluminescence technique (IMMULITE 2000, Siemens Healthcare Sector, Erlangen, Germany) with an LLOQ of 0.02 nmol/L and mean intraassay and interassay CVs < 10%. Calculated free T was assessed by Vermeulen’s formula [13], using the ISSAM online calculator (http://www.issam.ch/freetesto.htm, accessed on 15 January 2023). A default albumin level of 4.3 g/dL was used for this calculation. We also calculated free E_2_ levels from their total levels and SHBG concentration. LH and FSH were measured in a single assay using an automated immunochemiluminescence method (Architect^®^ FSH, Architect^®^ LH, Abbot Ireland diagnostics Division, Lisnamuck, Longford, Co. Longford, Ireland).

### 2.3. Assessment of Cardiovascular Autonomic Function: Ewing’s Score and Power Spectral Heart Rate Data

Cardiovascular autonomic function was assessed by the tests proposed by Ewing et al. [14], and recommended by the American Diabetes Association’s consensus statement on standardized measures for individuals with diabetes [15]. A detailed description of the methodology is available in our previous studies [5]. CAN was detected using the two currently available gold standard methods: (a) the standardized cardiac autonomic reflex tests (CARTs) described by Ewing et al. in 1970 [14]; and (b) power spectral HR variability by analyzing beat-to-beat intervals from short-duration electrocardiogram recordings. We used a modification of the Ewing score to diagnose the presence of CAN, which scored the responses of HR variability to deep breathing (E/I ratio), standing (30:15 ratio), and Valsalva’s maneuver (VAL ratio), and the response of BP (∆SBP) to active standing as normal (0 points), borderline (0.5 points), or positive (1 point). A composite score ≥ 1 was diagnostic of CAN. We classified CAN as early or mild when the Ewing’s score was between 1 and 2, or as definite when the score was ≥2.

We assessed HR variability using a Monitor VitalScan Medeia^®^ System device (Santa Barbara, CA, USA). All patients fasted, except for basal insulin. They abstained from food, nicotine, caffeine, and certain medications for 12 h before testing. Serum glucose was checked to rule out hypoglycemia, with no patient having levels <70 mg/dL.

Adrenergic innervation assessed BP and HR changes 5 min after standing. A difference of ≤10 mmHg indicated normal, 11–29 mmHg borderline, and ≥30 abnormal results. Orthostatic hypotension was defined as a >20 mmHg systolic BP drop. Resting HR was measured by palpating the radial pulse, with HR > 100 beats per minute considered tachycardia.

Power spectral HR data were obtained from 10 min EKG recordings using VitalScan Medeia^®^ software HW7-HWWW6T. This method used the Fourier method, which transformed R–R intervals into wavelets, differentiating low frequency (LF) 0.04–0.15 Hz (sympathetic and parasympathetic influence) and high frequency (HF) 0.15–0.4 Hz (parasympathetic activity).

### 2.4. Proton Nuclear Magnetic Resonance Spectroscopy Metabolomics

Serum samples were shipped to Biosfer Teslab in dry ice for the quantification of LMWM by proton nuclear magnetic resonance (^1^H-NMR) spectroscopy. Serum samples (200 μL) were previously diluted with 50 µL deuterated water and 300 µL of 50 mM phosphate buffer solution (PBS) at pH 7.4, consisting of 30.70 Na2HPO4 mM and 19.30 NaH2PO4 mM, before analysis.

^1^H-NMR spectra were recorded at 300 K on a Bruker Avance III 600 spectrometer (Bruker Biospin, Rheinstetten, Germany), operating at a proton frequency of 600 MHz. One-dimensional ^1^H pulse experiments were carried out using 1D Carr–Purcell–Meiboom–Gill (cpmg) spectra. A total of 64 transients were collected into 64k data points for each spectrum. The acquired spectra were phased, baseline-corrected, and referenced before performing the automatic metabolite profiling of the spectra dataset through an adaptation of Dolphin [16].

### 2.5. Statistical Analysis

We show data as means ± SD or median (IQR) according to their distribution, and counts (percentages), in addition to their 95% confidence interval (CI) (lower limit; upper limit) when appropriate. To ensure normality before using parametric tests, we applied logarithmic transformations to all skewed variables.

To explore the impact of the physiological decrease in sex steroids, particularly in women during the menopausal transition, we categorized our patient sample into subgroups based on age below or above 50 years. We chose this cut-off because this is the median age of natural menopause in Caucasian women [17]. We also defined menopause according to the Stages of Reproductive Aging Workshop (STRAW) staging system developed from data from multiple longitudinal cohort studies [18], considered the gold standard for characterizing reproductive aging. Women were divided into two groups: (i) women of reproductive age and (ii) women in late perimenopause (characterized by amenorrhea > 60 days plus a circulating FSH > 25 IU/l) or menopause (defined retrospectively after 12 months of amenorrhea).

For the analysis of continuous and discrete variables, univariate two-way general linear models (GLM) or binary logistic regression analyses were employed. We used logistic regression analysis to estimate the association of LMWM (including those variables significant in the univariate analysis) with the presence/absence of CAN adjusted for the clinical variables, introducing sex (coded as 0 = women and 1 = men), age (years), duration of type 1 diabetes, A_1c_ levels, microvascular complications (coded as 0 = absent and 1 = present) and BMI as independent variables. We examined the correlation between LMWM and Ewing’s autonomic function test score using Spearman’s correlation analysis.

Statistical significance was set at a *p*-value < 0.05. The analyses were performed using SPSS Statistics 23 (SPSS Inc., Chicago, IL, USA).

## 3. Results

### 3.1. Sex-Based Clinical and Biochemical Characteristics of Patients with Cardioautonomic Neuropathy

A total of 323 subjects were included (46% females, median age of 42 ± 19 years and median duration of diabetes 18 ± 19 years). According to the STRAW staging system, forty-five women were postmenopausal. Demographics and clinical features of the whole population of patients with T1D are summarized in Table 1.

A total of 30 patients (9.3%) were obese. As expected, obese patients had reduced eGDR compared with non-obese patients (8.09 ± 2.02 vs. 9.29 ± 1.93 mg/kg/min, *p* = 0.006). Additionally, men displayed lower eGDR and required larger total daily insulin doses. We observed differences in body fat distribution and lipid metabolism between women and men, with females showing higher mean values of fat mass, total cholesterol, and HDL cholesterol concentrations compared to males. Conversely, men exhibited higher values of BMI, waist circumference, waist-to-hip ratio, and systolic and diastolic BP. Men showed better glycemic control as indicated by lower A_1c_ levels. Regarding hormonal parameters, men had significantly lower levels of FSH and LH than women did. As expected, men had higher total T, free T, total T/total E_2_, and free T/free E_2_ molar ratios compared with women, while women had higher total E_2_, free E_2_, and SHBG concentrations than men.

The overall prevalence of CAN was 28% [95% CI: 23; 33]. When considering all subjects as a whole, CAN prevalence (defined as an Ewing’s score >1) was not significantly different between women and men [32% (25; 40) vs. 24% (19; 31), respectively, *p* = 0.132]. However, when age was taken into account, menopause resulted in an excess risk of CAN in women. In women with perimenopause or menopause (*n* = 47), the prevalence of CAN doubled that of younger women [vs. 51% (37; 65) vs. 23% (16; 32), respectively, *p* < 0.001], with an OR 3.5 (1.7; 7.2) of having CAN compared with their reproductive-aged counterparts. Regardless of sex, patients with CAN were older, had a longer duration of diabetes, and presented micro- and macroangiopathy more frequently, compared with those without CAN.

Table 1 summarizes the clinical and biochemical characteristics of patients both when considering all subjects as a whole or in women and men separately, and categorized by the presence or absence of CAN. We observed statistically significant interactions between sex and CAN. Women with T1D and CAN showed higher total T/E_2_ and free T/E_2_ molar ratios, and lower E_2_ and free E_2_, compared to those without CAN. Conversely, men with T1D and CAN exhibited lower free T, total T/E_2_, and free T/E_2_ molar ratios, and higher total and free E_2_ compared to those without CAN.

### 3.2. Association between Metabolomic Intermediate Metabolism with CAN

**Figure 1:**
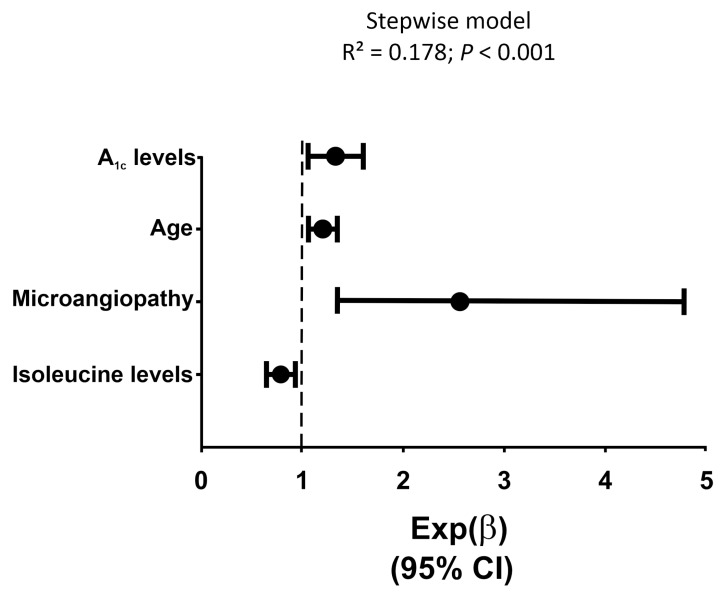
Logistic regression analysis estimating the association of low molecular weight metabolites with the presence/absence of cardioautonomic dysfunction, adjusted for clinical variables such as sex (coded as 0 = women and 1 = men), age (years), duration of type 1 diabetes, insulin doses, A_1c_ levels, microvascular complications (coded as 0 = absent and 1 = present), and BMI as covariates. Closed circles are Exp(B) values and bars and whiskers represent their 95% confidence intervals (95% CI).

Table 2 summarizes the results of the univariate analysis comparing LMWM levels of patients with and without CAN. Patients with CAN showed lower levels of valine, isoleucine, and threonine, and higher lactate levels, than subjects without CAN. These differences remained after adjusting for BMI and eGDR (isoleucine 29 (17) vs. 33 (17); *p* = 0.003; valine, 200 ± 37 vs. 214 ± 48, *p* = 0.003; and threonine, 228 ± 50 vs. 242 ± 51, *p* = 0.007); Subsequently, we conducted a binary logistic regression analysis to estimate the association between LMWM and CAN. After adjusting for clinical variables, the logistic regression model (R^2^ Nagelkerke = 0.178, *p* < 0.001) retained isoleucine [Exp(β) = 0.972 (95%IC 0.952; 0.003)], age [Exp(β) = 1.031 (95%IC 1.010; 1.053)], A_1c_ levels [Exp(β) = 1.361 (95%IC 1.058; 1.752)], and microangiopathy [Exp(β) = 2.560 (95%IC 1.372; 4.778)] as the main determinants of CAN (Figure 1).

### 3.3. Correlation of LMWM Profile with Tests of Cardiovascular Autonomic Function

Table 3 shows the correlations between LMWM profiles and autonomic dysfunction indices. In summary, valine and isoleucine showed positive correlations with E/I ratio and VAL index, and negative correlations with total Ewing’s score. Threonine showed positive correlations with VAL index and ∆SBP. Conversely, lactate displayed negative correlations with the VAL index, normalized low frequency, and normalized high frequency, along with positive correlations with total Ewing’s score.

### 3.4. Interactions of Age, Sex, and CAN on LMWM Levels

Table 4 shows the LMWM levels among age-based subgroups. Patients over 50 years of age, considering men and women as a whole, displayed higher levels of creatinine, lactate, and the amino acids alanine, glycine, glutamate, glutamine, and tyrosine. Furthermore, we observed an interaction between sex and age concerning acetate and glycerol, with increased concentrations in women and decreased concentrations in men over age 50. Regardless of age, men exhibited lower levels of creatine, glycerol, and glycine, and higher levels of acetone and creatinine, compared with women. In terms of amino acids, men showed lower levels of glycine and higher levels of glutamate, glutamine, isoleucine, leucine, threonine, and valine compared to women.

We also performed an interaction analysis between LMWM with CAN, sex, and menopausal status (in women) or age (in men). The results of the analysis are shown in Table S1. We observed an interaction between postmenopausal status and CAN with respect to glutamine. Glutamine levels decreased only in postmenopausal women with CAN.

Finally, Table 5 provides a summary of the effects of age, sex, CAN, and their interactions on the levels of the metabolites studied here.

## 4. Discussion

Our objective was to investigate the relationship between circulating levels of low molecular weight metabolites in patients with CAN and T1D, emphasizing the effects of sexual dimorphism and age. To our knowledge, this study is the first to apply 1H-NMR spectroscopy for low molecular weight metabolite profiling in T1D patients with CAN, while simultaneously considering the influences of sex and age within a single metabolomic analysis. The main findings revealed by this study consist of an association between CAN with decreased isoleucine, valine, and threonine levels in individuals with T1D, which appeared regardless of the sex and age of the subjects. Although disturbances in amino acid levels are believed to contribute to the development of diabetes and its complications, their role in the pathogenesis of these disorders remain unclear [19,20]. Alterations in protein balance, dietary intake, amino acid transport across cell membranes, and increased gluconeogenesis in the liver and kidneys are undoubtedly important factors in this process [19].

The metabolic profiles associated with diabetes risk and the disease itself encompass metabolites beyond glucose metabolism. In patients with T1D, levels of leucine, isoleucine, valine, phenylalanine, and tyrosine are elevated, while levels of glycine, glutamate, and threonine are reduced when compared to both matched controls and insulin-treated patients. However, when treated with insulin via a euglycemic clamp, these metabolic differences were eliminated. This indicates that the metabolome is highly sensitive to the presence or absence of insulin in T1D, potentially driving diabetes complications. The increase in glucogenic amino acids compared to control subjects suggests an altered metabolism of these metabolites in T1D, despite overall diabetes control [21].

In general, available evidence supports that an increase in plasma branched-chain amino acid (BCAA) levels such as isoleucine and valine are present in patients with T1D, type 2 diabetes, and their chronic complications [19,22,23,24,25]. The association of elevated plasma BCAA levels with obesity, first made in the 1960s by Felig et al. [25], has been confirmed by multiple investigators [22,23,25]. More importantly, emerging studies suggest a causal role of BCAAs in the pathogenesis of obesity and insulin resistance [26]. Increased BCAA concentrations in T1D is explained by amination of branched-chain keto acids in visceral adipose tissue and decreased uptake of BCAAs by the muscle [27]. Regarding the chronic complications of diabetes, Rojas et al. [24] found that the amino acids valine, isoleucine, and leucine increased more than twofold from 12 weeks post-STZ in animal models of diabetes and peripheral neuropathy.

Surprisingly, even after adjustment for multiple variables (especially those related to insulin-resistance), the decrease in isoleucine levels remained a determinant of the presence of CAN in our subjects with T1D. Why, in the case of CAN, do BCAA levels decrease instead of increasing as previously described in other animal and human models of diabetes or peripheral diabetic neuropathy? A possible hypothesis that would explain our results, as well as others previously described in patients with T1D and CAN [9], relies on the regulation of amino acid metabolism at the central nervous system level.

Although the molecular catabolic pathway of BCAAs has been well delineated, the physiological mechanisms that regulate BCAA degradation and, hence, determine circulating BCAA levels, remained elusive [28]. A previous report in an animal model concluded that that insulin signaling in the hypothalamus is mandatory for lowering plasma BCAA levels, most probably by inducing hepatic BCAA catabolism [29]. Moreover, Gannaban et al. [30] reported that acute stimulation of vagal motor neurons in the dorsal motor nucleus was sufficient to decrease plasma BCAA. These findings suggest a critical role of insulin signaling in neurons for BCAA regulation, and raise the possibility that this control may be mediated primarily via vagal outflow [30]. Therefore, the induction of parasympathetic outflow through stimulation of vagal motor neurons represents a potential mechanism for BCAA regulation. We hypothesize that in patients with CAN, the predominance of sympathetic tone may lead to the observed decrease in plasma BCAA levels. Activation of the sympathetic nervous system such as that occurring in the systemic inflammatory response syndrome and in hypermetabolic states has been postulated as one of the possible causes of decreased BCAA levels [19,27]. In agreement with our present results, previous research has indicated lower levels of methionine and isoleucine in patients with T1D and CAN [9]. Moreover, lower baseline glutamine levels correlate with CAN and were also associated with the progression of CAN at 3 years after adjustment for baseline A_1c_, blood glucose, BMI, cholesterol, urine microalbumin-to-creatinine ratio, estimated glomerular filtration rate, and years of diabetes. Therefore, significant changes in the anaplerotic flux in the TCA cycle could be the critical defect underlying CAN progression [21]. In our analysis, glutamine levels were decreased only in postmenopausal women with CAN (Table 3). Another explanation for our present results could be that deficiencies in specific amino acids could adversely affect the autonomic nervous system, potentially due to neuropathy-induced muscle wasting, implying a catabolic state in these patients [9].

Table 5 provides an overview of the LMWM differences observed among the subgroups of 323 patients with T1D. Our findings also revealed sexual dimorphism in the plasma metabolomic profiles of individuals with T1D, regardless of the presence of absence of CAN. Men exhibited higher levels of various branched and aromatic amino acids, which are characteristic of obesity-associated metabolic dysfunction. Specifically, men with T1D had elevated levels of glutamate, glycine, isoleucine, leucine, and threonine, suggesting possible sex-based metabolic differences in amino acid turnover or muscle metabolism. These sex-related changes are in conceptual agreement with those obtained from non-diabetic subjects, where most amino acids that were increased in men were also found at higher levels in women with functional hyperandrogenism and obesity [31,32,33]. Additionally, our findings are consistent with the larger rates of muscular protein synthesis during training observed in men when compared to women [34]. This sexual dimorphism did not appear to be a consequence of any direct association with obesity, as there were no differences in BMI between sexes in this study [34]. Moreover, serum lipid profiles were similar in men and women, aside from the expected lower HDL cholesterol concentrations of men. Therefore, other factors such as genetic differences, sex hormone concentrations, insulin resistance, and body composition (such as waist circumference and fat mass, which were increased in male individuals in our study) might cause these sex specific differences in metabolomic profiles.

Interestingly, age influenced the impact of sex on LMWM profiles. For some metabolites associated with metabolic dysfunction, such as glycerol and acetate, age exacerbated these profiles in women but not in men. In other words, age worsened metabolomic profiles in women with T1D, particularly after menopause, while men appeared to be protected to some extent from the negative effects of age on the metabolome. Additionally, a few statistically significant interactions between age and CAN on LMWM were observed: patients with CAN under 50 years showed decreased acetate levels, while those over 50 years exhibited increased tyrosine levels, regardless of sex.

We acknowledge several limitations in our study that warrant consideration when interpreting present findings. Firstly, the cross-sectional design of our study limits our ability to establish causality. Secondly, our study applied a targeted metabolomics approach, focusing on a specific subset of metabolites in serum. This limited scope may restrict our ability to comprehensively understand the broader metabolomics landscape, as the blood metabolome may not necessarily reflect tissue-specific abnormalities. Lastly, the generalizability of our results may be limited, since participants were not randomly recruited, potentially introducing a selection bias. Despite these limitations, our study benefitted from a relatively large sample size of individuals with well-controlled T1D, thereby enhancing the statistical robustness of our findings. Additionally, the utilization of a gold standard technique for the comprehensive exploration of the autonomic cardiovascular system contributed to a thorough understanding of the physiological mechanisms under investigation, increasing the scientific reliability of our study.

In conclusion, our present findings underscore the heterogeneity of the metabolic alterations underlying CAN among patients with T1D. Considering sex and age is crucial given the complex interplay of these variables with metabolism, highlighting the importance of incorporating these factors in metabolomics investigations. Conversely, the alteration of metabolites affected by insulin resistance despite strict glycemic control highlights the importance of conducting a comprehensive assessment of metabolic status of patients with T1D that extends beyond glucose level control.

## Figures and Tables

**Table 1:** Clinical, biochemical, and hormonal characteristics of patients with T1D, categorized by the presence or absence of cardioautonomic neuropathy (CAN) when considered as a whole and in women and men separately.

	All Patients	Women			Men			CAN	Sex	CAN*Sex
Variable	All	All women	no CAN	CAN	All men	no CAN	CAN			
	(*n* = 323)	(*n* = 147)	(*n* = 100)	(*n* = 47)	(*n* = 176)	(*n* = 133)	(*n* = 43)			
Clinical										
Age, years	42 (19)	41 (21)	38 (20)	47 (26)	43 (19)	40 (21)	46 (15)	***<0.001***	0.791	0.883
Never smokers [N (%)]	190 (59)	86 (59)	59 (59)	27 (57)	104 (59)	82 (62)	22 (51)	0.225	0.55	0.469
Antiaggregant therapy [N (%)]	39 (12)	15 (10)	4 (4)	11 (23)	24 (14)	13 (10)	11 (26)	***0.011***	0.855	0.259
Statin therapy [N (%)]	118 (37)	46 (31)	27 (27)	19 (40)	72 (41)	48 (36)	24 (56)	***0.024***	0.146	0.701
On antihypertensives [N (%)]	50 (15)	23 (16)	10 (10)	13 (28)	27 (15)	17 (13)	10 (23)	0.102	0.633	0.429
Body mass index, kg/m^2^	24 (5)	24 (5)	24 (6)	23 (4)	25 (5)	25 (5)	26 (5)	0.158	0.002	0.061
Obesity [N (%)]	30 (9)	16 (11)	11 (11)	5 (11)	14 (8)	7 (5)	7 (16)	0.141	0.989	0.116
Waist circumference, cm	84 (17)	77 (16)	77 (16)	78 (14)	88 (16)	87 (16)	93 (15)	***0.041***	***<0.001***	0.134
Hip, cm	99 (12)	98 (15)	98 (17)	101 (10)	100 (11)	100 (10)	101 (14)	0.482	0.504	0.834
Waist to hip ratio	0.85 (0.14)	0.79 (0.10)	0.78 (010)	0.80 (0.13)	0.90 (0.12)	0.89 (0.11)	0.94 (0.12)	0.054	***<0.001***	0.124
Fat mass (%)	24 ± 10	30 ± 8	30 ± 8	30 ± 7	18 ± 8	17 ± 7	21 ± 8	***0.041***	***<0.001***	0.052
Systolic blood pressure, mmHg	120 (17)	117 (19)	115 (16)	121 (24)	122 (13)	121 (13)	128 (20)	***0.001***	***<0.001***	0.947
Diastolic blood pressure, mmHg	77 (15)	73 (13)	73 (13)	74 (15)	78 (15)	78 (14)	80 (18)	0.101	***<0.001***	0.443
Diabetes										
Age at diagnosis of T1D, years	18 (16)	19 (18)	16 (18)	22 (17)	17 (13)	17 (13)	18 (17)	***0.009***	0.531	0.514
Duration of T1D, years	18 (19)	17 (18)	15 (158)	19 (20)	20 (20)	18 (20)	23 (18)	***0.012***	0.756	0.283
DKA at diagnosis [N (%)]	119 (37)	52 (35)	33 (33)	19 (40)	67 (38)	54 (41)	13 (30)	0.226	0.314	0.139
CSII [N (%)]	79 (25)	42 (29)	29 (29)	13 (28)	37 (21)	26 (20)	11 (26)	0.413	0.824	0.464
Total insulin dose, units/day	40 (24)	34 (20)	34 (20)	32 (17)	47 (23)	45 (24)	48 (17)	0.785	0.001	0.614
Daily insulin dose, units/kg/day	0.56 (0.25)	0.55 (0.23)	0.55 (0.23)	0.50 (0.24)	0.57 (0.24)	0.56 (0.26)	0.62 (0.21)	0.554	0.895	0.896
eGDR, mg/kg/min	9.6 (2.7)	10.3 (2.2)	10.5 (2.1)	9.8 (3.4)	9.1 (2.4)	9.3 (2.1)	8.2 (2.1)	***0.002***	***0.002***	0.907
Microangiopathy [N (%)]	66 (20)	31 (21)	15 (15)	16 (34)	35 (20)	19 (14)	16 (37)	***0.002***	0.754	0.735
Macroangiopathy [N (%)]	16 (5)	7 (5)	3 (3)	4 (9)	9 (5)	4 (3)	5 (12)	***0.038***	0.624	0.743
Metabolic parameters										
A_1c_ (%)	7.2 (1.3)	7.3 (1.7)	7.2 (1.4)	7.8 (1.2)	7.1 (1.0)	7.0 (1.0)	7.4 (1.1)	0.002	***0.029***	0.669
A_1c_, mmol/mol	55 (14)	56 (19)	55 (15)	62 (13)	54 (11)	53 (11)	57 (12)	0.002	***0.025***	0.655
Total cholesterol, mmol/L	4.5 ± 0.82	4.7 ± 0.81	4.7 ± 0.75	4.6 ± 0.91	4.3 ± 0.80	4.2 ± 0.75	4.5 ± 0.91	0.678	***0.005***	0.092
HDL cholesterol, mmol/L	1.48 (0.47)	1.68 (0.60)	1.68 (0.52)	1.65 (0.83)	1.37 (0.39)	1.40 (0.39)	1.32 (0.46)	0.208	***<0.001***	0.356
LDL cholesterol, mmol/L	2.57 ± 0.65	2.60 ± 0.68	2.65 ± 0.68	2.50 ± 0.68	2.54 ± 0.63	2.48 ± 0.58	2.72 ± 0.75	0.593	0.733	***0.02***
Triglycerides, mmol/L	0.66 (0.31)	0.63 (0.32)	0.61 (0.31)	0.69 (0.45)	0.68 (0.32)	0.65 (0.32)	0.72 (0.24)	***0.011***	0.053	0.748
eGFR, mL/min/1.73 m^2^	89 (20)	84 (15)	86 (11)	81 (20)	94 (22)	94 (22)	88 (19)	***0.001***	***<0.001***	0.109
Hormonal parameters										
FSH, IU/L *	4.6 (4.4)	6.9 (45.0)	5.8 (15.9)	32.6 (57.2)	3.5 (2.9)	3.4 (2.9)	4.4 (3.9)	***0.001***	***<0.001***	0.229
LH, IU/L *	3.8 (4.4)	7.1 (17.8)	5.5 (16.1)	14.5 (18.3)	3.2 (2.0)	3.0 (2.1)	3.6 (2.0)	***<0.001***	***<0.001***	0.211
Total T, nmol/L *	15 (23)	1.2 (0.7)	1.0 (0.7)	1.1 (0.8)	23 (11)	23 (11)	22 (10)	0.94	***<0.001***	***0.06***
Total E_2_, pmol/L *	99 (94)	218 (370)	250 (358)	89 (344)	90 (115)	87 (38)	102 (45)	***0.026***	***0.046***	***0.001***
Total T/ E_2_ molar ratio *	177 (254)	7 (16)	5 (11)	12 (49)	249 (123)	258 (135)	222 (100)	***0.019***	***<0.001***	***<0.001***
SHBG, nmol/L *	62 (43)	87 (54)	83 (51)	100 (61)	50 (28)	49 (28)	51 (29)	***0.023***	***<0.001***	0.668
Calculated free T, pmol/L *	272 (395)	10 (7)	11 (8)	8 (6)	381 (173)	392 (185)	350 (117)	0.114	***<0.001***	0.126
Calculated free E_2_, pmol/L *	1.8 (1.6)	3.0 (5.2)	3.8 (4.6)	1.3 (4.4)	1.7 (0.8)	1.6 (0.9)	1.9 (0.6)	***0.026***	***0.046***	***0.001***
Calculated free T/E_2_, molar ratio *	157 (239)	4 (10)	3 (7)	8 (30)	255 (109)	244 (115)	190 (90)	***0.014***	***<0.001***	***<0.001***

* 19 women taking hormonal contraceptives were excluded. Abbreviations: BP, blood pressure; CSII, continuous subcutaneous insulin infusion; DKA, diabetes ketoacidosis; eGDR, estimated glucose disposal rate; eGFR, estimated glomerular filtration rate MDRD-4 formula); E_2_, estradiol; FSH, follicle-stimulating hormone; HDL, high density-lipoprotein; LDL, low density-lipoprotein; LH, luteinizing hormone; SHBG, sex hormone-binding globulin; T, testosterone, T1D, type 1 diabetes. Data of continuous variables are shown as means ± SD or median (interquartile range) according to their distribution. The differences in continuous variables among groups were analyzed by univariate two-way general linear models (GLM). *p*-values (bold indicate significant, <0.05).

**Table 2:** Low molecular weight metabolites categorized by the presence of cardioautonomic neuropathy (CAN).

	All Patients (*n* = 323)	No CAN (*n* = 233)	CAN (*n* = 90)	*p*
Energy and Homeostasis Metabolites				
Acetate	23 (15)	23 (14)	25 (13)	0.086
Acetone	20 (21)	22 (23)	18 (16)	0.339
Creatine	27 (20)	27 (20)	26 (18)	0.381
Creatinine	62 (19)	62 (18)	64 (22)	0.137
Glucose	7055 (4131)	6952 (3745)	7631 (5649)	0.452
Glycerol	132 (72)	133 (69)	131 (77)	0.809
Lactate	308(159)	302(136)	362 (233)	***0.006***
Hydroxybutyrate	25 (66)	27 (80)	20 (48)	0.530
Amino Acids				
Alanine	310 (82)	307 (74)	326 (91)	0.063
Glycine	218 (69)	217 (59)	230 (97)	0.178
Glutamate	66 (27)	65 (26)	69 (28)	0.427
Glutamine	434 ± 58	432 ± 54	439 ± 66	0.289
Histidine	77 ± 12	76 ± 12	77 ± 13	0.569
Isoleucine	32 (17)	33 (17)	29 (17)	***0.002***
Leucine	96 (27)	99 (28)	93 (29)	0.178
Threonine	238 ± 51	242 ± 51	228 ± 50	***0.033***
Tyrosine	37 ± 9	36 ± 9	37 ± 10	0.285
Valine	210 ± 45	214 ± 48	200 ± 37	***0.014***

Data are arbitrary units and are expressed as mean ± SD or median (interquartile range). *p*-values (bold indicate significant, <0.05).

**Table 3:** Correlations between associated low molecular weight metabolites and cardioautonomic test values.

	∆SBP	E/I Ratio	VAL Index	30:15 Index	Normalized Low Frequency	Normalized High Frequency	Score Ewing Total
Lactate	ρ = −0.057 *p* = 0.314	ρ = −0.083 *p* = 0.150	***ρ = −0.136*** ***p = 0.018***	ρ = −0.014 *p* = 0.801	***ρ = −0.173*** ***p = 0.002***	***ρ = −0.164*** ***p = 0.004***	***ρ = 0.163*** ***p = 0.004***
Isoleucine	ρ = 0.085 *p* = 0.129	***ρ = 0.135*** ***p = 0.017***	***ρ = 0.156*** ***p = 0.006***	ρ = 0.080 *p* = 0.156	ρ = 0.067 *p* = 0.239	ρ = 0.054 *p* = 0.343	***ρ = −0.158*** ***p = 0.004***
Threonine	***ρ = 0.127*** ***p = 0.024***	ρ = 0.096 *p* = 0.093	***ρ = 0.146*** ***p = 0.011***	ρ = 0.004 *p* = 0.943	ρ = 0.069 *p* = 0.224	ρ = 0.012 *p* = 0.829	ρ = −0.109 *p* = 0.052
Valine	***ρ = 0.124*** ***p = 0.026***	***ρ = 0.123*** ***p = 0.030***	***ρ = 0.206*** ***p < 0.001***	ρ = 0.035 *p* = 0.535	ρ = 0.065 *p* = 0.248	ρ = 0.024 *p* = 0.675	***ρ = −0.146*** ***p = 0.009***

Spearman’s Rho correlation coefficients and *p*-value between pairs of variables are provided. *p*-values (bold indicate significant, <0.05). Abbreviations: E/I, expiration/inspiration; ∆SBP, response in systolic blood pressure to orthostatism; VAL, Valsalva.

**Table 4:** Low molecular weight metabolites considering all patients with type 1 diabetes as a whole and as a function of sex and age.

Variable	Women			Men					
	All (*n* = 147)	≤50 years (*n* = 103)	>50 years (*n* = 44)	All (*n* = 176)	≤50 years (*n* = 135)	>50 years (*n* = 41)	Sex	Age	Sex*Age
Energy and Homeostasis Metabolites									
Acetate	22 (14)	19 (13)	28 (15)	25 (17)	24 (18)	25 (13)	0.397	0.131	***0.018***
Acetone	18 (23)	18 (22)	23 (31)	26 (22)	27 (22)	22 (18)	***0.040***	0.271	0.397
Creatine	30 (18)	27 (19)	31 (23)	24 (19)	25 (18)	24 (22)	***0.002***	0.806	0.196
Creatinine	54 (12)	53 (12)	58 (13)	69 (16)	68 (14)	73 (20)	***<0.001***	***<0.001***	0.806
Glucose	7597 (4606)	7368 (4648)	8159 (5141)	7336 (3936)	7729 (4573)	7049 (3519)	0.438	0.127	0.154
Glycerol	142 (64)	138 (63)	143 (96)	136 (79)	149 (80)	117 (68)	***0.001***	0.786	***0.028***
Lactate	313 (183)	307 (187)	351 (198)	308 (138)	301 (125)	336 (155)	0.451	***0.034***	0.307
Hydroxybutyrate	27 (95)	27 (71)	29 (139)	31 (72)	35 (80)	13 (52)	0.808	0.781	0.709
Amino Acids									
Alanine	314 (92)	306 (91)	319 (98)	306 (68)	297 (73)	318 (67)	0.339	***0.023***	0.706
Glycine	227 (81)	217 (78)	255 (52)	209 (53)	207 (48)	220 (65)	***0.001***	***<0.001***	0.053
Glutamate	61 (27)	62 (28)	61 (25)	70 (28)	67 (29)	78 (29)	***<0.001***	***0.022***	0.411
Glutamine	416 ± 58	408 ± 57	438 ± 57	445 ± 53	442 ± 53	455 ± 21	***0.001***	***0.004***	0.118
Histidine	76 ± 12	76 ± 13	75 ± 10	78 ± 13	77 ± 13	79 ± 11	0.153	0.858	0.200
Isoleucine	28 (15)	28 (16)	27 (10)	35 (17)	35 (17)	36 (17)	***<0.001***	0.073	0.865
Leucine	92 (26)	91 (27)	93 (21)	106 (30)	108 (30)	102 (29)	***<0.001***	0.767	0.204
Threonine	220 ± 46	218 ± 48	224 ± 41	257 ± 47	259 ± 50	250 ± 38	***<0.001***	0.856	0.167
Tyrosine	36 ± 9	36 ± 9	34 ± 10	37 ± 9	36 ± 9	41 ± 9	0.228	***<0.001***	0.462
Valine	198 ± 44	193 ± 43	196 ± 36	226 ± 44	228 ± 47	220 ± 36	***<0.001***	0.750	0.228

Data (μmol/L) are shown as means ± SD or median (interquartile range) according to their distribution. The differences in continuous variables among groups were analyzed by univariate two-way general linear models (GLM). *p*-values (bold indicate significant, <0.05).

**Table 5:** Overview of the changes in low molecular weight metabolites observed in patients with type 1 diabetes.

Metabolite	Sex (Men vs. Women)	Age (≤50 vs. >50 Years Old)	Interaction Sex*Age	CAN (No CAN vs. CAN)	Interaction CAN*Age
Energy and Homeostasis Metabolites					
Acetate	=	=	↓ in men >50 years↑ in women >50 years	=	↓ CAN only in >50 years
Acetone	↑ in men	=	=	=	=
Creatine	↑ in women	=	=	=	=
Creatinine	↑ in men	↑ in >50 years	=	=	=
D-glucose	=	=	=	=	=
Glycerol	↑ in women	=	↓ in men >50 years↑ in women >50 years	=	=
Hydroxybutyrate	=	=	=	=	=
Lactate	=	↑ >50 years	=	↑ in CAN	=
Amino Acids					
Alanine	=	↑ >50 years	=	=	=
Glutamine	↑ in women	↑ >50 years	=	=	=
Glutamate	↑ in men	↑ >50 years	=	=	=
Glycine	↑ in men	↑ >50 years	=	=	=
Histidine	=	=	=	=	=
Isoleucine	↑ in men	=	=	↓ in CAN	=
Leucine	↑ in men	=	=	=	=
Threonine	↑ in men	=	=	↓ in CAN	=
Tyrosine	=	↑ >50 years	=	=	↑ CAN only in >50 years
Valine	↑ in men	=	=	↓ in CAN	=

Abbreviations: CAN, cardiac autonomic neuropathy. Up arrows (↑) indicate elevated levels; down arrows (↓) indicate decreased levels.

## Data Availability

The data that support the findings of this study are available from the corresponding author, [LN-C], upon reasonable request. The data are not publicly available because they containing information that could compromise the privacy of research participants.

## Supplementary Materials

The following supporting information can be downloaded at: https://www.mdpi.com/article/10.3390/metabo14080436/s1, Table S1: Low molecular weight metabolites categorized by the presence of cardioautonomic neuropathy (CAN), and as a function of sex and age (men) or menopausal stage (women).
