# Supplementary material for: Investigating the Link between Intermediate Metabolism, Sexual Dimorphism, and Cardiac Autonomic Dysfunction in Patients with Type 1 Diabetes
Source: Metabolites. 2024 Aug 6;14(8):436. doi: 10.3390/metabo14080436 (PMC11356535; doi:10.3390/metabo14080436)
Supplement: Supplementary file 1 [file metabolites-14-00436-s001.zip › metabolites-3120312-supplementary.pdf]

Supplemental material

Table S1. Low molecular weight metabolites categorized by the presence of cardioautonomic neuropathy (CAN), and as a function of sex and age (men) or menopausal stage (women).

	**No CAN (*n* = 233)**				**CAN (*n* =90)**				***p***
	**Women (*n* = 100)**		**Men (*n*= 133)**		**Women (*n*=47)**		**Men (*n*=43)**		
	Premenopausal (*n* = 78)	Postmenopausal (*n* = 22)	Men ≤ 50 yrs (*n* = 107)	Men >50 yrs (*n* = 26)	Premenopausal (*n* = 24)	Postmenopausal (*n* = 23)	Men ≤ 50 yrs (*n* = 28)	Men >50 yrs (*n* = 15)	
**Energy and Homeostasis Metabolites**									
Acetate [c]	20 ± 9	30 ± 14	27 ±14	25 ± 12	24 ± 14	27 ± 9	33 ± 28	25 ± 10	***<0.005***
Acetone	29 ± 45	29 ± 23	31 ± 22	29 ± 17	29 ± 25	21 ± 16	29 ± 17	17 ± 7	ns
Creatine [a]	29 ± 13	42 ± 24	27 ± 15	26 ± 11	32 ± 22	34 ± 20	24 ± 12	26 ± 17	***<0.001***
Creatinine [a]	53 ± 10	58 ± 22	67 ± 11	76 ± 12	62 ± 38	60 ± 12	72 ± 13	73 ± 14	***<0.001***
Glucose [d]	6780 ± 3014	8397 ± 2817	7294 ± 3165	7414 ± 2250	8330 ± 4136	7691 ± 3127	8109 ± 4005	5971 ± 1956	***<0.005***
Glycerol [a, b]	142 ± 62	145 ± 68	134 ± 58	118 ± 42	142 ± 59	154 ± 101	134 ± 57	112 ± 46	***<0.005***
Lactate	354 ± 156	331 ± 112	314 ± 142	403 ± 208	354 ± 156	385 ± 182	406 ± 139	377 ± 155	ns
Hydroxybutyrate	87 ± 202	91 ± 129	57 ± 68	51 ± 59	72 ± 121	43 ± 81	56 ± 73	35 ± 55	ns
**Amino Acids**									
Alanine	317 ± 67	346 ± 96	305 ± 58	321 ± 44	339 ± 103	327 ± 70	323 ± 65	333 ± 76	ns
Glycine [a, b]	220 ± 42	265 ± 57	213 ± 41	228 ± 46	232 ± 81	270 ± 66	212 ± 42	231 ± 43	***<0.001***
Glutamate [a]	62 ± 19	72 ± 54	72 ± 21	77 ± 21	56 ± 19	71 ± 25	81 ± 26	76 ± 21	***<0.001***
Glutamine [a, e, f]	400 ± 51	453 ± 44	444 ± 51	444 ± 51	437 ± 77	424 ± 63	448 ± 63	456 ± 53	***<0.005***
Histidine	76 ± 12	74 ± 9	76 ± 12	80 ± 12	79 ± 17	73 ± 10	80 ± 13	77± 9	ns
Isoleucine [a, d]	32 ± 13	28 ± 9	41 ± 19	38 ± 10	28 ± 10	30 ± 12	35 ± 11	30 ± 13	***<0.005***
Leucine [a]	94 ± 24	94 ± 16	109 ± 26	106 ± 19	88 ± 15	93 ± 18	109 ± 27	98 ± 18	***<0.001***
Threonine [a]	220 ± 47	218 ± 35	260 ± 54	255 ± 34	210 ± 50	226 ± 46	244 ± 53	233 ± 41	***<0.001***
Tyrosine [b, e]	36 ± 10	33 ± 6	36 ± 9	40 ± 8	34 ± 10	41 ± 10	34 ± 9	43 ± 10	***<0.005***
Valine [a, d]	194 ± 42	192 ± 37	229 ± 50	229 ± 34	182 ± 35	196 ± 37	218 ± 37	198 ± 23	***<0.005***

Data are arbitrary units and are expressed as mean ± SD. The level of statistical significance was set a *p* value < 0.05. Comparisons among groups were performed by an univariate two-way GLM (adjusted for diabetes duration and A_{1c} levels).[a] Significant differences between men and women; [b] Significant differences among older/younger patients (men) or menopausal stage (women) independently of sex; [c] Statistically significant interaction between sex and group of age (men) or menopausal stage (women).[d] Statistically significant interaction between diagnosis of CAN.[e] Statistically significant interaction between diagnosis of CAN and group of age (men) or menopausal stage (women).[f] Statistically significant interaction between sex, group of age (men) or menopausal stage (women) and diagnosis of CAN.
